# Therapeutic Irrigant Procedures for Treating Apical Periodontitis (TIPTAP): A triple‐blinded parallel‐group randomized controlled phase I/II trial

**DOI:** 10.1111/iej.14233

**Published:** 2025-04-14

**Authors:** Satnam Singh Virdee, Nasir Zeeshan Bashir, Melissa M. Grant, Paul R. Cooper, Phillip L. Tomson

**Affiliations:** ^1^ Institute of Clinical Sciences, School of Dentistry & Birmingham Dental Hospital University of Birmingham Birmingham UK; ^2^ MRC Biostatistics Unit University of Cambridge Cambridge UK; ^3^ Department of Oral Sciences, Faculty of Dentistry University of Otago Dunedin New Zealand

**Keywords:** apical periodontitis, biomarkers, cone beam computed tomography, dentine extracellular matrix components, postoperative pain, regenerative endodontics

## Abstract

**Background:**

Solubilized endogenous dentine extracellular matrix components (dECMs) are potent mediators in pulp regeneration and could potentially promote similar healing effects in diseased periradicular tissues by upregulating local mesenchymal stem cell‐derived regenerative events.

**Aims:**

(1) Determine if endodontic treatment outcomes with irrigation regimes promoting dECM release (17% ethylenediaminetetraacetic acid [EDTA]) are equivalent to conventional regimes (5.25% sodium hypochlorite [NaOCl]) in mature permanent teeth with asymptomatic apical periodontitis. (2) Explore changes in pain scores, expressions of periradicular tissue fluid (PTF)‐derived inflammatory mediators, and volumtric changes in lesion size between the different irrigant regimes.

**Methods:**

Forty single‐rooted teeth, from 37 healthy adults, were block randomized into parallel groups of irrigation with either 17% EDTA, optimized for dECM solubilization, or 5.25% NaOCl (*n* = 20). All other aspects of the endodontic protocol were standardized over two visits with 14 days of calcium hydroxide intracanal medicament. Patient‐reported pain scores were recorded at six hours and then daily for one week post‐instrumentation and post‐obturation. PTF samples were collected pre‐instrumentation and pre‐obturation, where analyte profiles (pg/TPC) were determined using an O‐link Target‐48 cytokine array. Treatment outcomes were clinically and radiographically assessed with cone beam computed tomography at 1 year using dichotomous criteria (favourable/unfavourable) based on volumetric change in lesion size. Participants, operators and assessors were blinded, and per‐protocol analyses were conducted using binary logistic regression models with initial alpha values for statistical comparisons set at *p* < .05.

**Results:**

A 90% recall rate was achieved at one year (NaOCl: 19; EDTA: 17). Favourable outcomes were observed in 89.5% of treatments using NaOCl and 94.1% of treatments using EDTA irrigation, with median lesion volume reductions of 92.5% (IQR: 67.33–99.13) and 95.84% (IQR: 78.81–100), respectively, (*p* > .05). Odds of unfavourable periradicular healing with EDTA irrigation were 0.53 [95% CI: 0.04–6.44; *p* > .05]. No serious adverse effects or atypical pain patterns were reported, although two acute exacerbations occurred post‐instrumentation with NaOCl irrigation (*p* > .05). Target‐48 panels consistently detected 15 inflammatory analytes in both groups (CCL‐2, ‐3, ‐4; CSF‐1; CXCL‐8; HGF; IL‐1β, ‐6, ‐18; MMP‐1, ‐12; OLR‐1; OSM; TNFSF‐10; VEGF‐A), all of which reduced pre‐obturation. At this stage, IL‐6 and ‐18 were significantly more abundant in the intervention group (*p* < .05).

**Conclusions:**

Therapeutic irrigant regimes promoting dECM solubilization resulted in one‐year treatment outcomes equivalent to conventional irrigant protocols with no serious adverse effects reported.

## INTRODUCTION

Apical periodontitis is an inflammatory condition of the periodontium affecting 52% of the global adult population (Tibúrcio‐Machado et al., 2021). It is initiated when endodontic micro‐organisms stimulate the host response, a process mediated by numerous auto‐ and para‐crine signalling molecules that are detectable within periradicular tissue fluid (PTF; Nair 2004; Márton & Kiss, 2014, Virdee et al., 2019). Current therapeutic strategies focus solely on the microbial component of this dynamic equilibrium, where a pro‐healing micro‐environment is established via chemomechanical debridement. Whilst this antimicrobial strategy may enable a highly co‐ordinated sequence of periradicular regenerative events, of which local mesenchymal stem cell (MSC) play a significant role, this approach provides no direct biological stimulus (Lin & Rosenberg, 2011). This could explain why, although there have been advances in technology and materials, clinical outcomes for root canal treatment in necrotic teeth with periapical radiolucencies have remained relatively stagnant for five decades. More specifically, apical periodontitis remains present in approximately one‐quarter of infected teeth (Gulabivala & Ng, 2023; Ng et al., 2008). This could also account for why success rates decrease in larger lesions (Ng et al., 2011). Therefore, persistent periapical lesions may not only represent an inability to reduce the endodontic microbial load below a currently unknown critical threshold but also an inadequate capacity to simulate physiological regeneration (Siqueira Jr & Rôças, 2008). A failure to improve predictability in the treatment of periradicular disease has resulted in significant interest in the development of more biologically driven treatment strategies for apical periodontitis, where greater emphasis is placed on utilizing local MSC‐based regenerative events that lead to periradicular tissue healing (Kim et al., 2018).

In recent years, a novel niche of dental MSCs has been identified from within human periradicular granulation tissue (Liao et al., 2011). In vitro, these periapical lesion‐derived MSCs (PL‐MSC) have demonstrated tremendous immunosuppressive and regenerative capabilities with strong commitment towards an osteogenic lineage (Dokić et al., 2012; Liao et al., 2011; Marrelli et al., 2013). For these reasons, these cells have been implicated as the key determinants of the periapical healing process following root canal treatment. Their presence, therefore, provides clinical opportunities to directly enhance local tissue regenerative events in mature permanent teeth via a cell‐free homing approach (Chrepa et al., 2015; Lyu et al., 2022; Virdee et al., 2022). This highly translatable approach, thus far only utilized in pulp regeneration of immature teeth, would involve upregulating PL‐MSC in situ by way of stimulating damaged periradicular tissues with signalling molecules responsible for co‐ordinating physiological healing events (Kim et al., 2018).

Notably, there is an abundant reservoir of odontoblast‐derived growth factors, cytokines, chemokines and other signalling molecules locally sequestered within the dentine's extracellular matrix during tooth development (Smith et al., 2012). Key examples include transforming growth factor‐beta 1 [TGF‐β1], often found at the highest abundance and used as a surrogate quantitative marker for this cocktail of molecules and their inherent bioactivity (Graham et al., 2006, Sadaghiani et al., 2016, Tomson et al., 2017); as well as bone morphogenetic protein [BMP]; vascular endothelial growth factor [VEGF]; and insulin growth factor [IGF] amongst many others (Jágr et al., 2012; Park et al., 2009; Widbiller et al., 2019). Proteoglycan bonds formed following dentinogenesis preserve these fossilized morphogens within the dentine; however, they can be released under appropriate local tissue conditions, and their bioactive properties are clinically harnessed using common endodontic irrigants (Baker et al., 2009; Galler et al., 2015). Of the various solutions thus far investigated, the chelating action, neutral pH and protease‐inhibiting properties of ethylenediaminetetraacetic acid (EDTA) currently make it the gold standard for releasing dentine matrix protein bioactivity (Tavares et al., 2021). The yield can be further enhanced when EDTA is ultrasonically activated; however, associated bioactivity becomes significantly reduced or even completely eliminated when dentine is pre‐treated by even relatively weak concentrations of sodium hypochlorite (NaOCl; Galler et al., 2015, Widbiller et al., 2017). Whilst re‐instrumentation could debride some of the dentine penetrated by NaOCl, which can range from 38 to 411 μm (Virdee et al., 2020), this can further weaken the tooth structure and still results in considerably less dentine matrix components being solubilized than when EDTA is exclusively used (Widbiller et al., 2022). This outcome can be attributed to its nonspecific proteinaceous properties that make the most commonly administered irrigant in endodontics incompatible with the integrity of local tissue bioactive molecules. The resulting EDTA‐derived dentine tissue extracts, nevertheless, termed dentine extracellular matrix components (dECM), demonstrate a potent capacity to promote regenerative activities within multiple endodontic MSC niches (Virdee et al. 2022). Similar effects were also recently demonstrated in PL‐MSCs (unpublished data). Furthermore, the synergistic activity of this cocktail exhibits a greater potency than the application of single recombinant morphogens; with their endogenous nature also overcoming many ethical issues associated with clinically using exogenous substitutes (Bègue‐Kirn et al., 1992; Lee et al., 2015; Widbiller et al., 2018).

To date, these previous findings have translated into the development of novel therapies for managing deep carious lesions and the exposed pulp as well as providing proof of concept for the cell‐free homing approaches to treating apical periodontitis (Duncan et al., 2019; Murray et al., 2002; Smith et al., 2016). Consequently, it has been hypothesized that therapeutic irrigant regimes optimized to liberate dECMs into root canals and the periradicular tissues thereafter could upregulate PL‐MSCs bioactivity to promote tissue healing in mature permanent teeth diagnosed with apical periodontitis (Virdee et al., 2022). At the time of conducting the present investigation, there were no prior interventional clinical studies that had tested this hypothesis.

## AIMS

The primary aim of this triple‐blinded parallel‐group randomized controlled phase I/II trial was to determine if the treatment outcomes with respect to periapical healing (O) of root canal therapy using conventional irrigant regimes (C; 5.25% NaOCl) were equivalent to those promoting the release of dECMs (I; 17% EDTA) in mature permanent teeth diagnosed with a necrotic pulp and asymptomatic apical periodontitis (P). Secondary objectives included exploring the longitudinal differences in (1) patient‐reported pain scores post‐instrumentation and post‐obturation; (2) PTF‐derived inflammatory mediator concentrations and (3) periapical lesion volume change between irrigant regimes. The null hypothesis tested was that there were no significant differences in endodontic success rates between conventional irrigant regimes and those that promote the release of dECMs.

## MATERIALS AND METHODS

### Ethics

**FIGURE 1:**
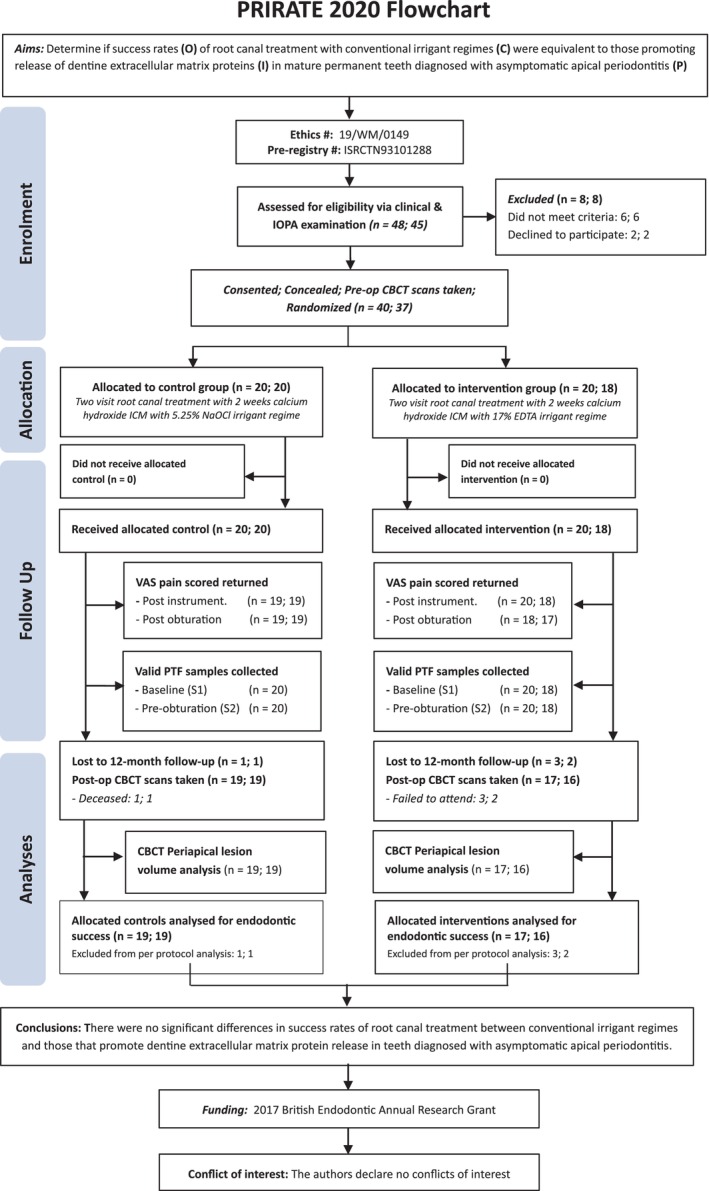
PRIRATE Flow Chart. Sample sizes represent participants at the tooth and patient level (t; p).

This interventional study was granted ethical approval from the National Health Service West Midlands Research Ethics Committee (reference no. 19/WM/0149) and reported in accordance with the 2019 Preferred Reporting Items for Randomized Trials in Endodontics (PRIRATE) guidelines (Nagendrababu et al., 2019; Figure 1). The protocol was registered *a priori* on the International Standard Randomized Controlled Trial Number (ISRCTN) Registry (ISRCTN93101288). Informed written consent was obtained from volunteering patients at their initial consultation following the provision of detailed verbal and written information on the purposes, methods and risks. This was confirmed again via a telephone conversation 1 week later, after which consent forms were stored in the site file and the referring practitioner informed of participation by written correspondence.

### Study design and setting

A triple‐blinded parallel‐group randomized controlled phase I/II trial of equivalence with a 1:1 allocation ratio was conducted in the Endodontic Department at the Birmingham Dental Hospital between October 2019 and March 2024. The rationale for this phase I/II design was clinical risk management and to explore how the disease process responded to this relatively novel intervention, for which there has been no prior published clinical safety or efficacy data. A public and patient involvement group consisting of five non‐dental members who had undergone root canal treatment provided input into the planning and design of the study. There were no methodological amendments after the trial commenced.

### Participant selection criteria

Medically fit consenting adults (≥18 years) referred to the Endodontic Department at the Birmingham Dental Hospital for root canal treatment in mature permanent single‐rooted teeth diagnosed with a necrotic pulp and asymptomatic apical periodontitis were consecutively recruited. The exclusion criteria consisted of individuals who were: unwilling or unable to consent, pregnant or breast feeding; had undergone antimicrobial therapy 3 months prior to screening; immunocompromised (i.e. diabetes mellitus, human immunodeficiency virus, leukaemia, neutropenia, undergoing chemo‐ or systemic corticosteroid therapy); had altered bone metabolism (i.e. bisphosphonate or monoclonal antibody therapy, head and neck radiotherapy). Teeth that were multirooted; symptomatic; previously accessed or root filled; exhibited clinical signs and symptoms consistent with pulpitis or periapical abscess; had periodontal pocketih ≥ 5 mm within the same sextant; unable to retain a rubber dam; had apices that were immature or closely associated with the maxillary sinus; presented evidence of internal or external root resorption or vertical and horizontal root fractures were also excluded.

### Preoperative assessment

All diagnostic assessments were performed by a single operator (SSV). Briefly, a comprehensive pain, medical and dental history was taken to confirm the absence of symptoms, aetiology of disease and potential medical contra‐indications to participation. This was followed by a systematic extra‐ and intra‐oral hard and soft tissue examination where the selected tooth was subjected to a focused visual (restorability; restoration presence, type and quality; presence of caries, cracks and fractures), periapical (tenderness to percussion or palpation; presence of sinus or swelling), periodontal (six‐point probing depths, mobility) and occlusal (static and dynamic) assessment. This was supplemented with thermal (Endo‐Frost; Roeko) and electric (SybronEndo; Sybron Dental Specialities Inc.) pulp testing with reference to a healthy comparable contralateral tooth.

All potentially eligible teeth underwent digital long cone periapical (LCPA) radiographic assessment. These were obtained via a paralleling technique with rectangular collimation using phosphor plate sensors (Dürr Dental) positioned intra‐orally with Rinn short arm (Dentsply Sirona) aiming devices to improve standardization. Phosphor plates were then exposed at 70 kV, 7.0 mA and 0.08 sec. exposure time with a wall‐mounted intra‐oral x‐ray unit (Heliodent Plus; Dentsply Sirona) prior to being processed using the VistaScan system (Dürr Dental). Teeth with a necrotic pulp and asymptomatic apical periodontitis were defined as those that were clinically unresponsive to pulp, percussion or palpation testing but radiographically demonstrated a periradicular radiolucency with no associated sinus (Glickman, 2009).

After confirming eligibility and attaining informed consent, a baseline limited field of view (4 × 4 cm) cone beam computed tomograph (CBCT; 3D Accuitomo 170; J Morita) was acquired. Operating parameters were set at 85 kV, 4.5 mA and 17.5 s. exposure time with a 360° arc of rotation and 250 μm voxel size. Volumes were reconstructed to slice thicknesses of 1 mm and reformatted for clinical use on Enterprise Imaging Software (AGFA Healthcare).

### Sample size

A smaller sample of 40 teeth (*n =* 20 per group) was selected for this phase I/II trial based on clinical safety and risk mitigation of the proposed irrigant regime, for which there was no prior clinical safety or efficacy data. This is nevertheless consistent with similarly designed studies published within the fields of endodontic tissue engineering (Arslan et al., 2019; Brizuela et al., 2020; Jha et al., 2019; Xuan et al., 2018) and the minimum criteria specified for inclusion within the European society of Endodontology (ESE) S3‐level clinical practice guidelines (Duncan et al., 2023).

### Randomization, concealment and blinding

Block randomization was performed at the tooth level using the online tool, Sealed Envelope (https://www.sealedenvelope.com/). A computer‐generated code list consisting of random permutated block sizes of four or six with a 1:1 allocation ratio was utilized and concealed from researchers. At the time of randomization, immediately prior to root canal treatment, the lead investigator (SSV) entered the participant identification number into the Sealed Envelope tool. The coded group allocation was then returned. The nature of the intervention was such that the operator, participant and assessors could all be blinded. More specifically, participants received root canal treatment with one of two clear irrigant solutions. These were coded by the operator and assessors, the former of whom delivered them into root canals via an identical regime with respect to volume, duration, activation and vehicle. In addition, a coded opaque bottle was used to store these irrigants; operator masks were fragranced with eucalyptus oil (Cerkamed) to disguise odours and plastic patient and bracket table coverings were used to prevent accidental NaOCl bleaching spots.

### Groups

The control group, which followed principles of a conventional anti‐microbial approach, consisted of performing root canal treatment exclusively using the 5.25% NaOCl (Cerkamed) irrigant solution. Conversely, the intervention group comprised solely of administering 17% EDTA (Cerkamed) throughout the procedure. This regime was informed by precursory in vitro methodological work‐up investigations that demonstrated intracanal solubilization and subsequent periradicular bioavailability of dentine extracellular matrix proteins (Virdee et al. [submitted]).

### Clinical procedure

All root canal procedures were performed by a single endodontically trained operator (SSV) under local anaesthesia (2% xylocaine and 1:80000 adrenaline; Dentsply Sirona) using a dental operating microscope (Global Surgical Corporation) and over two visits in accordance with ESE (2006) quality guidelines.

#### Visit 1

Crowns were initially stabilized by removing caries and any defective restorations were replaced (definitive direct restorations‐Clearfil Majesty; Kuraray Dental, Okayama, Japan/temporary indirect restorations‐Protemp; 3 M, Minesota, USA) when indicated. Teeth were subsequently isolated using rubber and liquid dams (Liquidam, Cerkamed) and traditional stable four‐walled access cavities were created using sterile cooled diamond burs under high‐volume aspiration. A 10/0.02 K‐Flex file (Dentsply Sirona) was then advanced down canals whilst connected to a Dentaport ZX electronic apex locator (J Morita, Osaka, Japan) to confirm patency and determine positions of the apical foramen and constriction as per the zero‐(i.e. terminal green bar) and 0.5‐markings (i.e. fifth green display bar), respectively (Connert et al. 2018). These were supplemented with LCPA radiographs and when file tips were ≥ 2 mm from the radiographic apex or extruded, length modifications were informed by a third electronic apex locator reading. Canals were then pre‐flared via crown‐down watch winding of 20/0.02 K‐flex files to the zero‐reading, dried using sterile 25/0.02 paper points set 2 mm short of this length, and patency filed with a 10/0.02 to disrupt dentinal debris apically and encourage intraradicular influx of tissue fluid. Baseline (S1) samples of PTF were then collected prior to any irrigation using an optimized paper point sampling protocol described previously (Virdee et al., 2023b). Briefly, three 15/.02 paper points (Classic, Unodent) were independently inserted into the canals until they were at a pre‐determined depth of 1 mm past the EAL's zero reading. These were maintained for 60 s before being removed. Wetted lengths were then measured with a 0.5 mm graduated stainless‐steel ruler under magnification after which cones were transferred into sterile microfuge tubes containing 250 μLs of phosphate buffered saline eluting buffer and stored at −80°C until analysis. Root canals were chemomechanically prepared in a crown‐down manner up to 40/.06 (Protaper Gold; Dentsply Sirona) at 300 rpm and 4 N torque. During instrumentation, 2 mL of 5.25% NaOCl (control) or 17% EDTA (intervention) solution was administered at room temperature between files using light steady finger pressure on a 27‐gauge side vented needle (Covidien, Dublin, Ireland) set 2 mm short of working length (WL). Thereafter, 5 mL of irrigant was deposited into canals and allowed to remain undisturbed for 4 min and 30 s before 30 s of passive ultrasonic irrigation with a 20/0.02 Irrisafe tip (Acteon) and Satelec (Acteon) at half power 1 mm from WL. This 5 min irrigant regime was repeated twice more prior to administering calcium hydroxide (UltraCal XS; Ultradent Products) intracanal medicament via a 27‐gauge side vented Navitip needle (Ultradent) set 2 mm from WL and temporarily sealing the access cavity with Kalzinol (Dentsply Sirona). If experienced, acute exacerbations of pain were managed by re‐irrigating canals with 15 mL of the assigned irrigant on a separate emergency visit.

#### Visit 2

After 14 days, teeth were isolated, re‐accessed and the calcium hydroxide intra‐canal medicament wicked away with sterile 25/0.02 paper points. The previously described 5‐minute irrigant regime was repeated three times prior to drying canals, collecting a second sample of PTF (S2), radiographically confirming master cone length and obturating with gutta‐percha and an epoxy resin‐based sealer (AH plus; Dentsply Sirona) via a continuous wave warm vertical condensation technique (B&L BioTech). Access cavities were immediately restored with a 2 mm subseal of bulk‐fill resin composite (SDR; Dentsply) followed by hybrid composite (Clearfil Majesty; Kuraray Dental). An immediate postoperative LCPA was taken and, where necessary, the patient's referring practitioner was asked to provide a definitive indirect cuspal coverage restoration.

### Treatment review

A 12‐month post‐treatment recall was conducted where teeth were clinically assessed and radiographically exposed to LCPA and CBCT's to review for signs and symptoms of active periradicular pathology. These procedures were conducted using the same methods and parameters described in the initial consultation.

## PRIMARY OUTCOME MEASURE

### Endodontic treatment outcome (*n*; %)

Outcomes for endodontic treatment were clinically and radiographically determined for both LCPA and CBCT imaging techniques using dichotomized loose [favourable vs. unfavourable] and strict [success vs. unsuccessful] criteria (Ng et al., 2011). Favourable outcomes were defined as those where teeth survived the recall interval and presented with an absence of symptoms and clinical signs of inflammation including tenderness to percussion, palpation, swellings or sinus tracts alongside radiographic evidence of complete or incomplete healing, which represented normal periodontal ligament space around the root or a reduction in lesion size, respectively. Success was defined as per the above description but exclusively with radiographic evidence of complete healing. Extractions for any reason following the initial treatment visit were considered unfavourable and unsuccessful in the final analyses.

Analyses were conducted by a consensus panel consisting of two endodontically trained assessors (SSV & NZB) who were experienced in interpreting LCPA and CBCT images over two face‐to‐face sessions. One appointment was dedicated to evaluating treatment outcomes based on LCPA radiographs and the second, taking place 4 weeks later, on CBCT scans, which were available in their entirety to align the vertical plane of roots centrally and parallel to the sagittal and coronal views. All images were randomized and viewed at optimal contrast and brightness via Enterprise Imaging Software (AGFA Healthcare) in a dimly lit room. Both assessors were blinded to treatment protocols and pre‐calibrated using 20 LCPA and 20 CBCT reference radiographs that were not part of the present study. These were identified by an oral and maxillofacial radiologist, represented teeth with or without periapical lesions and were used to confirm inter‐rater reliability of >90% prior to participant data analysis.

Follow‐up radiographs were scored against four possible periapical healing outcomes as described by Ng et al. (2011; complete, incomplete, uncertain and failure). Periapical lesions were defined as present when periapical radiolucencies were at least twice the width of the periodontal ligament space and considered reduced or increased when lesion length (mm) across the longest diameter or volume (mm^3^) changed by at least 20% (Low et al., 2008; Liang et al., 2014). The panel discussed discrepancies to arrive at a consensus and, in cases of disagreement, a third endodontically trained assessor (PLT) was consulted and arbitrated over the final decision.

## SECONDARY OUTCOME MEASURES

- **Patient‐reported pain scores** (*n*): To explore any differences in patient‐reported outcomes between irrigant regimes, a numerical rating scale (NRS) was used to evaluate the level of pain participants experienced at 6 h and then every 24 h up to 7 days post‐instrumentation (visit 1) and ‐obturation (visit 2). After visual and verbal explanation to facilitate use, individuals were instructed to report their discomfort by marking a point on a 10 cm line, anchored in two opposing extremes of “no pain” [0] and “pain as bad as it could be” [10], at the pre‐specified times. This was supplemented with a binary question relating to analgesic use. These numerical scores were then categorized into none [0]; mild [1–3]; moderate [4–6] and severe [7–10], which were presented as cumulative frequencies for each stratum per irrigant regime (Jalalzadeh et al., 2010).
- **Periradicular tissue fluid analysis** (pg/TPC): Coded baseline and pre‐obturation PTF samples were analysed using standard protocols previously described by Virdee et al.  (2023b) to explore if irrigant regimes differentially influenced periradicular inflammatory mediator profiles. Briefly, total volume (μL) was initially calculated via pre‐determined wetted length (mm) to volume (μL) standard curves followed by total protein concentration (TPC; μg/mL) using the Bradford dye‐binding assay (Thermo Fisher Scientific, MA, USA) against a standard series of bovine serum albumin with a plate reader set at a wavelength of 595 nm (Tecan Spark; Lifesciences, Switzerland). The Target‐48 Panel (O‐link, Uppsala, Sweden) was then utilized to quantify the concentration (pg/mL) of 45 different proteins related to the immune and inflammatory response within each sample. All assays were performed in duplicate as per manufacturer's instructions, with all outputs normalized to TPC and presented as pg/TPC. Inflammatory markers were considered absent and excluded from analyses when concentrations were below the lower limit of detection in > 25% of test PTF samples (Virdee et al., 2023b). Data points were assigned zero and maximum values when readings were below or above the respective limits of detection.
- **Percentage change in periapical lesion volume** (mm^3^; %): To explore differential rates of healing between irrigant regimes, three‐dimensional (3D) changes in periapical lesion volume (mm^3^) were determined independently by two pre‐calibrated assessors (SSV & NZB) using publicly available ITK‐Snap (V4.0.0) software and the semi‐automated methods described by Saini et al. (2023). First, periapical regions of test teeth within coded CBCT reconstructions were segmented across the axial, coronal and sagittal planes to encompass lesions. Secondly, upper and lower threshold limits were agreed upon amongst assessors and applied, after which the automated spherical filler “bubble” function was utilized to initialize the contour. Thirdly, this “bubble” was evolved repeatedly until the lesion was filled with manual adjustments made via the “paintbrush” function to eliminate over and under extensions. Thereafter, the software allowed this volume to be rendered in 3D and calculated in mm^3^ units. Resolved lesions were assigned a zero count with values from the two assessors averaged when within 2 mm^3^. Larger discrepancies however were resolved through discussion and combined re‐analysis until readings fell within this range. Percentage change in periapical lesion volume was then calculated for each case (i.e. percentage change in lesion volume [%] = [preoperative lesion volume–postoperative lesion volume]/preoperative lesion volume × 100).

### Statistical analysis

All coded data were statistically analysed per protocol in ‘R’ software (V.4.1.0; R Foundation for Statistical Computing, Vienna, Austria) by NZB. Interclass correlation coefficients (ICC) and Cohen's Kappa statistic were conducted to assess intra‐ and inter‐rater reliability for determining treatment outcome and periapical lesion volume, respectively. All paired and nonpaired categorical data comparisons were made with *McNemar's* and *Chi‐Squared* tests with data presented as frequencies alongside percentages. For quantitative data, an initial normality screen conducted via the Shapiro–Wilk test revealed a majority non‐normal distribution. Outcomes were thus presented as medians [interquartile range] with paired and nonpaired comparisons made with independent samples *Mann–Whitney U, Wilcoxon Matched Pair* and *Kruskal–Wallis* tests followed by *post hoc* pairwise comparison and Bonferroni family‐wise error rate correction with initial alpha values set at 0.05. Associations between the interventional protocol and an unfavourable treatment outcome using strict and loose criteria were investigated using generalized linear mixed models. These binary logistic regression models were fit using random effects to account for patient‐level clustering where more than one tooth was included per patient. Coefficients were exponentiated to obtain odds ratios (ORs), with respect to the conventional protocol, alongside corresponding 95% confidence intervals (CIs) and *p* values. Only after the completion of all statistical analyses was data uncoded.

## RESULTS

### Study characteristics

The present study spanned the period from October 2019 to March 2024, with participant recruitment conducted between October 2019 and February 2023 and reviews between January 2021 and March 2024. Participant enrolment, allocation, follow‐up and analyses are summarized in Figure 1. Overall, 40 teeth from 37 participants were enrolled in the trial after screening 48 potential candidates, of which 20 were randomized into the NaOCl and EDTA treatment groups, with one patient having two teeth enrolled in the two separate arms. All individuals were treated as intended; however, 4 teeth (NaOCl: 1; EDTA: 3) were lost to follow‐up as 1 patient deceased and 3 failed to return, leaving a recall rate of 90% (NaOCl:19; EDTA:17) per protocol analyses.

### Participant characteristics

The participant demographic and clinical characteristics at recall are summarized in Table 1. In brief, and at the tooth level, the median age was 36 years [25–43] (NaOCl: 37 [29–47]; EDTA: 36 [23–45]) with a 1:1 male‐to‐female ratio per group. Seven participants (NaOCl: 2; EDTA: 5) were of Afro‐Caribbean descent, 9 Asian (NaOCl: 4; EDTA: 5) and 20 Caucasian (NaOCl: 13; EDTA: 7). Seven (NaOCl: 5; EDTA: 2) teeth were from the mandible and 29 (NaOCl: 14; EDTA: 15) maxilla teeth, with incisors being the commonest tooth (33; NaOCl: 18; EDTA: 15) followed by canines (2; NaOCl: 1; EDTA: 1) and premolars (1; NaOCl: 0; EDTA: 1). Seven teeth presented coronally with direct restorations (NaOCl: 3; EDTA: 4) and 6 being indirect (NaOCl: 4; EDTA: 2) with the remainder being unrestored (23; NaOCl: 12; EDTA: 11). Traumatic dental injury was the most common indication for root canal treatment (16; NaOCl: 8; EDTA: 8) followed by iatrogenic injury (8; NaOCl: 4; EDTA: 4), idiopathy (8; NaOCl: 4; EDTA: 4), deep carious lesions (2; NaOCl: 1; EDTA: 1) and Ohler's class 1 dens‐in‐dente congenital abnormality (2; NaOCl: 2; EDTA: 0) with a median preoperative lesion volume of 74.6 mm^3^ (NaOCl: 78.9 [46.51–131.28]; EDTA: 66.8 [47.21–116.35]). Perioperatively, patency was achieved in all canals without perforations and all root fillings were of adequate quality and length; however, two teeth in the NaOCl group experienced an intra‐operative flare‐up and had to be scheduled for emergency appointments. At review, the median recall interval was 13 [12.0–14.0] months (NaOCl: 13 [12.0–13.5]; EDTA: 13 [12.0–14.0]) with the coronal seal in all teeth remaining intact. Both groups were statistically matched across all clinical and demographic characteristics (*p* > .05).

**TABLE 1:** Demographic and clinical characteristics.

Characteristics		Irrigant regime [*n* (%)]			
		Total (*n* = 36)	5% NaOCl (*n* = 19)	17% EDTA (*n* = 17)	*p* value^a^, ^b^
*Demographics*					
Age (years)	Median [IQR]	36 [25–43]	37 [29–47]	36 [23–45]	.754^a^
Sex	Female	18 (50.0)	10 (52.6)	8 (47.1)	.738^b^
	Male	18 (50.0)	9 (47.4)	9 (52.9)	
Ethnicity	Afro‐Caribbean	7 (19.4)	2 (10.5)	5 (29.4)	.213^b^
	Asian	9 (25.0)	4 (21.0)	5 (29.4)	
	Caucasian	20 (55.6)	13 (68.5)	7 (41.2)	
*Pre‐operative clinical factors*					
Inter‐arch position	Mandible	7 (19.4)	5 (26.3)	2 (11.8)	.271^b^
	Maxilla	29 (80.6)	14 (73.7)	15 (88.2)	
Intra‐arch position	Incisors	33 (91.7)	18 (94.7)	15 (88.3)	.558^b^
	Canine	2 (5.6)	1 (5.3)	1 (5.9)	
	Premolar	1 (2.8)	0 (0)	1 (5.9)	
Coronal restorations	NIL	23 (63.9)	12 (63.2)	11 (64.7)	.689^b^
	Direct	7 (19.4)	3 (15.8)	4 (23.5)	
	Indirect	6 (16.7)	4 (21.0)	2 (11.8)	
Disease aetiology	Deep Carious Lesion	2 (5.6)	1 (5.3)	1 (5.9)	.755^b^
	Congenital Abnormality	2 (5.6)	2 (10.5)	0 (0)	
	Iatrogenic	8 (22.2)	4 (21.0)	4 (23.5)	
	Trauma	16 (44.4)	8 (42.1)	8 (47.1)	
	Idiopathic	8 (22.2)	4 (21.0)	4 (23.5)	
Periapical lesion volume (mm^3^)	Median [IQR]	74.6 [46.51–131.28]	78.9 [49.81–183.30]	66.8 [47.21–116.35]	.363^a^
*Peri‐operative clinical factors*					
Patency achieved	Yes	36 (100)	19 (100)	17 (100)	1.00^b^
	No	0 (0)	0 (0)	0 (0)	
Perforations	Yes	0 (0)	0 (0)	0 (0)	1.00^b^
	No	36 (100)	19 (100)	17 (100)	
Inter‐appointment flare up	Yes	2 (5.6)	2 (10.5)	0 (0)	.169^b^
	No	34 (94.4)	17 (89.5)	17 (100)	
Root filling quality	Adequate	36 (100)	19 (100)	17 (100)	1.00^b^
	Inadequate	0 (0)	0 (0)	0 (0)	
Root filling length	>2 mm short	36 (100)	19 (100)	17 (100)	1.00^b^
	≤2 mm within	0 (0)	0 (0)	0 (0)	
	Extruded	0 (0)	0 (0)	0 (0)	
*Post‐operative clinical factors*					
Review interval (months)	Median [IQR]	13 [12.0–14.0]	13 [12.0–13.5]	13 [12.0–14.0]	.933^a^
Intact coronal seal	Yes	36 (100)	19 (100)	17 (100)	1.00^b^
	No	0 (0)	0 (0)	0 (0)	

^a^ Independent‐samples Mann–Whitney *U*.

^b^ Chi‐Squared test.

### Root canal treatment outcome

The intra‐ and inter‐rater agreements for determining treatment outcomes using LCPAs and CBCTs exceeded 0.90 for both categorical and dichotomous criteria, with results summarized in Table 2. All participants were clinically asymptomatic at recall. Based on CBCT imaging and loose criteria, favourable outcomes were observed across 16 (94.1%) tests and 17 (89.5%) control single‐rooted teeth, with odds of an unfavourable outcome in the former being 0.53 [95%CI: 0.04–6.44]. For strict criteria, success from CBCT analysis was observed in 8 (47.1%) test and 7 (36.8%) control teeth, with the odds of failure for the intervention being 0.66 [95% CI: 0.17–2.49] for 17% EDTA. When considering LCPAs, favourable outcomes were observed in 16 (94.1%) test and 18 (94.7%) control teeth, with the odds of an unfavourable outcome in the former being 1.13 [0.06–19.50]. Finally, for strict criteria in LCPAs, success was observed in 9 (52.9%) test and 11 (57.9%) control teeth, with odds of failure being 1.22 [95%CI: 0.33–4.56] for 17% EDTA. No significant differences were detected between the two irrigant regimes for LCPAs or CBCTs (*p* > .05).

**TABLE 2:** Outcome of root canal treatment in teeth diagnosed with asymptomatic apical periodontitis with conventional irrigant regimes and those that promote the release of dentine extracellular matrix proteins.

Imaging	Group	Loose criteria				Strict criteria			
		Favourable	Unfavourable	OR [95%CI]	*p* value^a^	Success	Failure	OR [95%CI]	*p* value^a^
LCPA	5.25% NaOCl	18 (94.7)	1 (5.2)	1	.936	11 (57.9)	8 (42.1)	1	.765
	17% EDTA	16 (94.1)	1 (5.9)	1.13 [0.06–19.50]		9 (52.9)	8 (47.1)	1.22 [0.33–4.56]	
CBCT	5.25% NaOCl	17 (89.5)	2 (10.5)	1	.619	5 (26.3)	14 (73.7)	1	.536
	17% EDTA	16 (94.1)	1 (5.9)	0.53 [0.04–6.44]		8 (47.1)	9 (52.9)	0.66 [0.17–2.49]	

^a^ 5% NaOCl success/favourable vs. 17% EDTA success/favourable [Chi Squared Test].

### Patient‐reported pain scores

**FIGURE 2:**
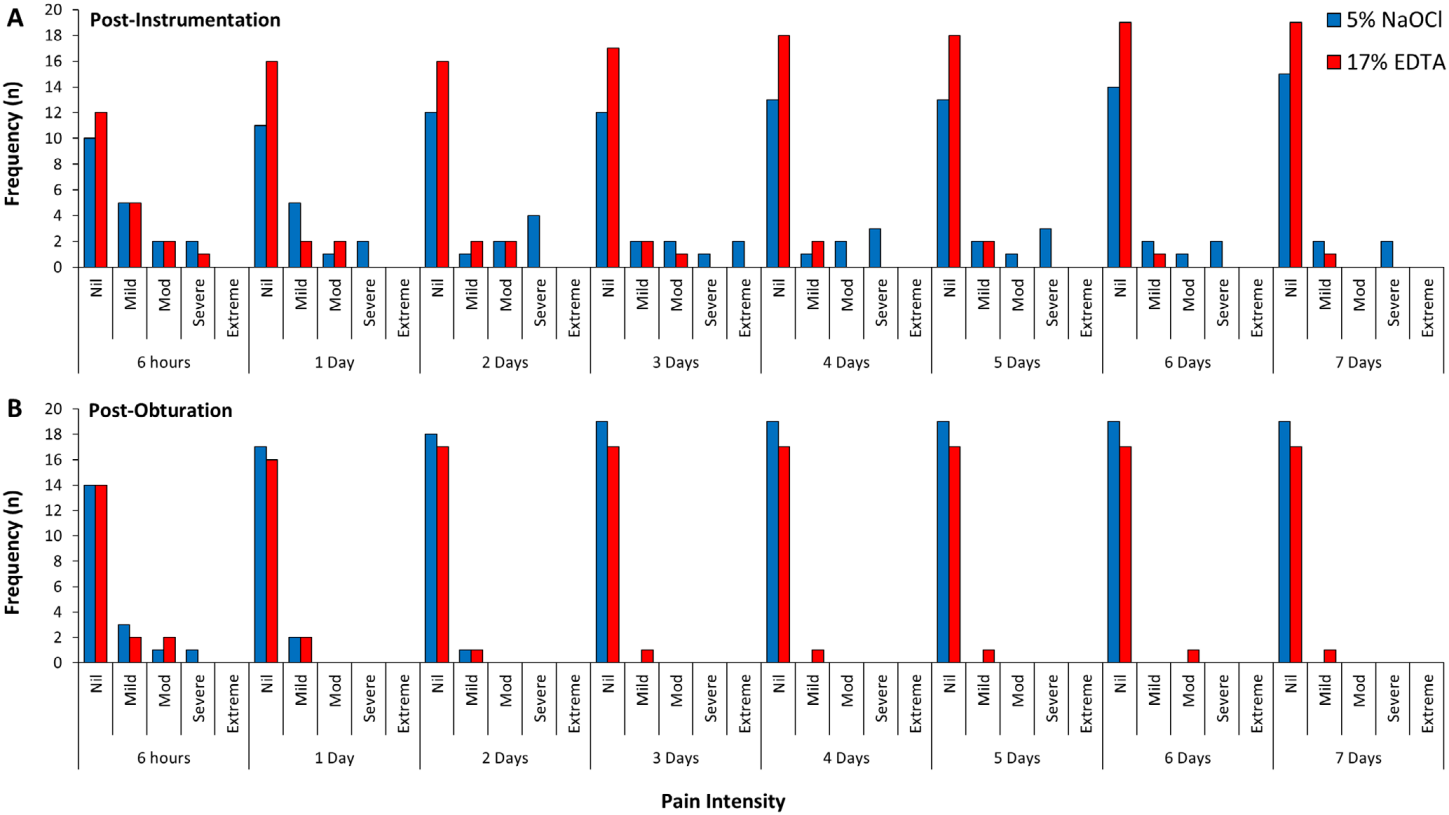
Pain intensity scores at several time points post‐instrumentation [a; 5.25% NaOCl *n* = 19; 17% EDTA *n* = 20] and ‐obturation [b; 5.25% NaOCl *n* = 19; 17% EDTA *n* = 18] following irrigation using conventional irrigant regimes (5.25% NaOCl) and those that promote the release of dentine extracellular matrix proteins (17% EDTA). Pain intensity categorized as No Pain = NRS 0; Mild = NRS 1–3; Mod = NRS 4–6; and Severe = NRS 7–10.

Post‐instrumentation, 19 (95%) and 20 (100%) participants within NaOCl and EDTA groups, respectively, returned valid NRS pain scores, whereas data from 19 (95%) control and 18 (90%) test patients were available post‐obturation, with results summarized in Figure 2. No inter‐group comparisons detected statistically significant differences at either treatment stage, with pain incidence in the former (NaOCl: 9 [47.4%]; EDTA: 11 [55.0%]) and latter (NaOCl: 5 [26.3%]; EDTA: 4 [22.2%]; *p* < .05). Notably, pain severity post‐instrumentation was milder to moderate in the EDTA group (8 [40.0%]) than NaOCl (4 [21.0%]), which was characterized by more severe discomfort (NaOCl: 5 [26.3%]; EDTA: 1 [5.3%]) for longer durations (NaOCl: 7 days; EDTA: 5 days) and greater use of analgesics (NaOCl: 8 [40.0%]; EDTA: 4 [20.0%]). This contrasted post‐obturation NRS scores for both groups, where there was less incidence (NaOCl: 5 [26.3%]; EDTA: 4 [22.2%]), milder severity (mild–moderate NaOCl: 3 [15.7%]; EDTA: 4 [22.2%]), shorter duration (NaOCl: 2 days; EDTA: 1 day) and less analgesic intake (NaOCl: 2 [10.5%]; EDTA: 2 [11.1%]). Participants most commonly experienced pain 6 hours after each treatment stage.

### Periradicular tissue fluid analysis

PTF samples were collected from all participants at each stage of treatment (100%) with results summarized in Table 3 and Appendix A and displayed in Appendix B. Whilst intra‐group comparisons revealed significant reductions in paper point wetted length, total absorbed volume and TPC from baseline to pre‐obturation (*p* < .001), there were no significant differences in these characteristics between groups (*p* > .05). The Target‐48 panel consistently detected 15 of a potential 45 analytes including chemokine ligand [CCL]‐2,‐3 and ‐4; colony stimulating factor [CSF]‐1; hepatocyte growth factor [HGF]; interleukin [IL]‐1β,‐6,‐8 [CXCL‐8] and ‐18; matrix metallopeptidase [MMP]‐1 and ‐12; oxidized low‐density lipoprotein receptor [OLR]‐1; oncostatin M [OSM]; tumour necrosis factor superfamily [TNFSF]‐10 and vascular endothelial growth factor [VEGF]‐A, all of which were similar in abundance between groups at baseline (*p* > .05). All analyte concentrations were reduced prior to obturation within both groups. The EDTA group had a statistically higher concentration of IL‐6 and IL‐18 than the control samples just prior to obturation (*p* < .05), where the most abundant cytokines for both groups were **MMP‐12** (NaOCl: 261.8 [22.00–520.40]; EDTA: 111.0 [12.36–460.78]), **CXCL‐8** (NaOCl: 105.2 [7.36–982.28]; EDTA: 84.69 [24.95–1845.91]) and **OLR‐1** (NaOCl: 43.0 [4.37–779.27]; EDTA: 57.3 [1.45–1614.18]).

**TABLE 3:** Longitudinal analysis of periradicular tissue fluid sampled from teeth diagnosed with asymptomatic apical periodontitis undergoing root canal treatment with conventional irrigant regimes and those that promote the release of dentine extracellular matrix components.

PTF characteristics	Baseline (S1)			Pre‐obturation (S2)		
	5% NaOCl	17% EDTA	*p* value^a^	5% NaOCl	17% EDTA	*p* value^a^
Total absorbed volume (μL)	1.8 [0.77–2.87]	2.0 [1.18–2.97]	.541	0.4 [0.26–0.83]^B^	0.5 [0.26–0.89]^b^	.698
Total protein concentration (μg/mL)	322.0 [243.23–719.08]	295.0 [255.39–501.74]	.841	183.6 [141.85–221.51]^B^	180.5 [131.20–255.07]^b^	.862
Analyte/total protein (pg/mg)						
Chemokine ligand [CCL]‐2	14.7 [5.35–43.86]	48.8 [12.11–230.61]	.134	3.9 [0.97–31.90]	8.0 [2.85–59.97]	.231
Chemokine ligand [CCL]‐3	12.1 [2.45–58.17]	18.9 [9.42–70.37]	.512	2.3 [0.55–11.69]	2.9 [0.64–15.31]^b^	1
Chemokine ligand [CCL]‐4	31.60 [5.59–165.31]	77.0 [18.73–267.87]	.289	5.16 [1.32–30.90]	6.9 [0.62–59.49]^B^	.947
Colony stimulating factor [CSF]‐1	2.9 [1.63–6.77]	4.5 [2.24–12.04]	.265	0.7 [0.02–5.19]	1.4 [0.57–4.96]^b^	.231
Hepatocyte growth factor [HGF]	119.6 [24.60–204.72]	221.4 [92.63–436.65]	.121	13.54 [2.32–35.32]^b^	35.6 [1.93–210.34]^b^	.265
Interleukin [IL]‐1β	148.7 [48.33–560.90]	550.8 [57.78–972.01]	.231	3.0 [0.256–7.68]^b^	4.7 [2.08–21.91]^B^	.142
Interleukin [IL]‐6	5.7 [2.75–18.39]	8.3 [3.88–15.04]	.779	0.2 [0.16–1.87]	3.8 [0.58–30.69]	.017*
Interleukin [CXCL]‐8	491.6 [73.74–3547.34]	1316.4 [222.77–6040.90]	.414	105.2 [7.36–982.28]	84.69 [24.95–1845.91]^b^	.799
Interleukin [IL]‐18	109.1 [29.2–202.75]	184.4 [102.81–394.74]	.277	1.4 [1.01–15.92]^b^	43.9 [10.80–223.81]	.030*
Matrix metallopeptidase [MMP]‐1	41.9 [6.46–143.86]	28.6 [9.14–136.87]	.947	2.5 [0.03–72.29]	10.16 [0.19–110.73]	.820
Matrix metallopeptidase [MMP]‐12	708.4 [43.10–4695.83]	380.8 [118.02–1320.01]	.718	261.8 [22.00–520.40]	111.0 [12.36–460.78]	.478
Oxidized low‐density lipoprotein Receptor [OLR]‐1	1190.8 [330.96–2647.97]	2237.8 [1347.68–3061.87]	.201	43.0 [4.37–779.27]^b^	57.3 [1.45–1614.18]^b^	.862
Oncostatin M [OSM]	25.4 [3.89–61.38]	51.86 [24.01–157.49]	.165	1.1 [0.21–11.61]^b^	0.9 [0.18–22.06]^b^	.947
Tumour necrosis factor superfamily [TNFSF]‐10	42.9 [10.01–88.10]	59.09 [37.53–205.17]	.134	0 [0–2.65]^b^	0 [0–17.49]^B^	.968
Vascular endothelial growth factor [VEGF]‐A	118.0 [31.34–190.53]	207.0 [88.86–408.76]	.091	23.3 [6.07–91.67]	22.6 [10.09–147.96]^b^	.512

^a^ Inter‐group comparison (NaOCl vs. EDTA); results outlined in *p* value subsections of baseline and pre‐obturation columns; *Independent Samples Mann–Whitney U* test.

^b^ Intra‐group comparison (baseline vs. pre‐obturation); [b] and [B] indicate *p* values of <.05 and <.01, respectively; results outlined in pre‐obturation columns for each irrigant; *Wilcoxon Matched Paired* test.

* *p* < .05.

### Percentage change in periapical lesion volume

**FIGURE 3:**
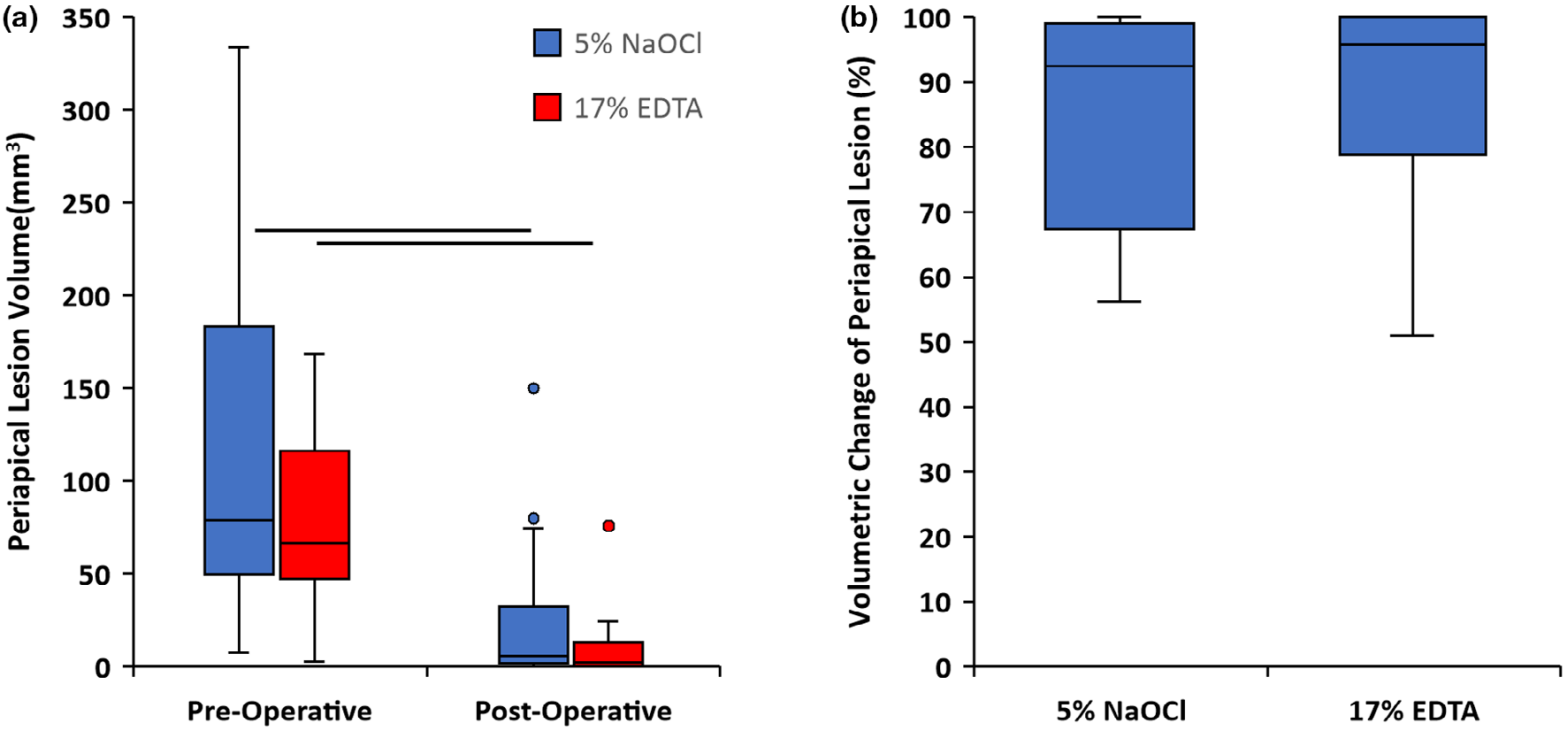
Volumetric (a) and percentage change (b) in periapical lesion size based on cone beam computed tomography scans of teeth diagnosed with asymptomatic apical periodontitis undergoing root canal treatment with conventional irrigant regimes and those that promote release of dentine extracellular matrix proteins. Data presented as box and whisker plots where central bars represent the median alongside upper and lower interquartile ranges at the edge of boxes and minimum and maximum values for the whiskers. Statistically significant comparisons within groups (*p* < .01; *Wilcoxon Matched Paired tests*) presented as horizontal lines.

**FIGURE 4:**
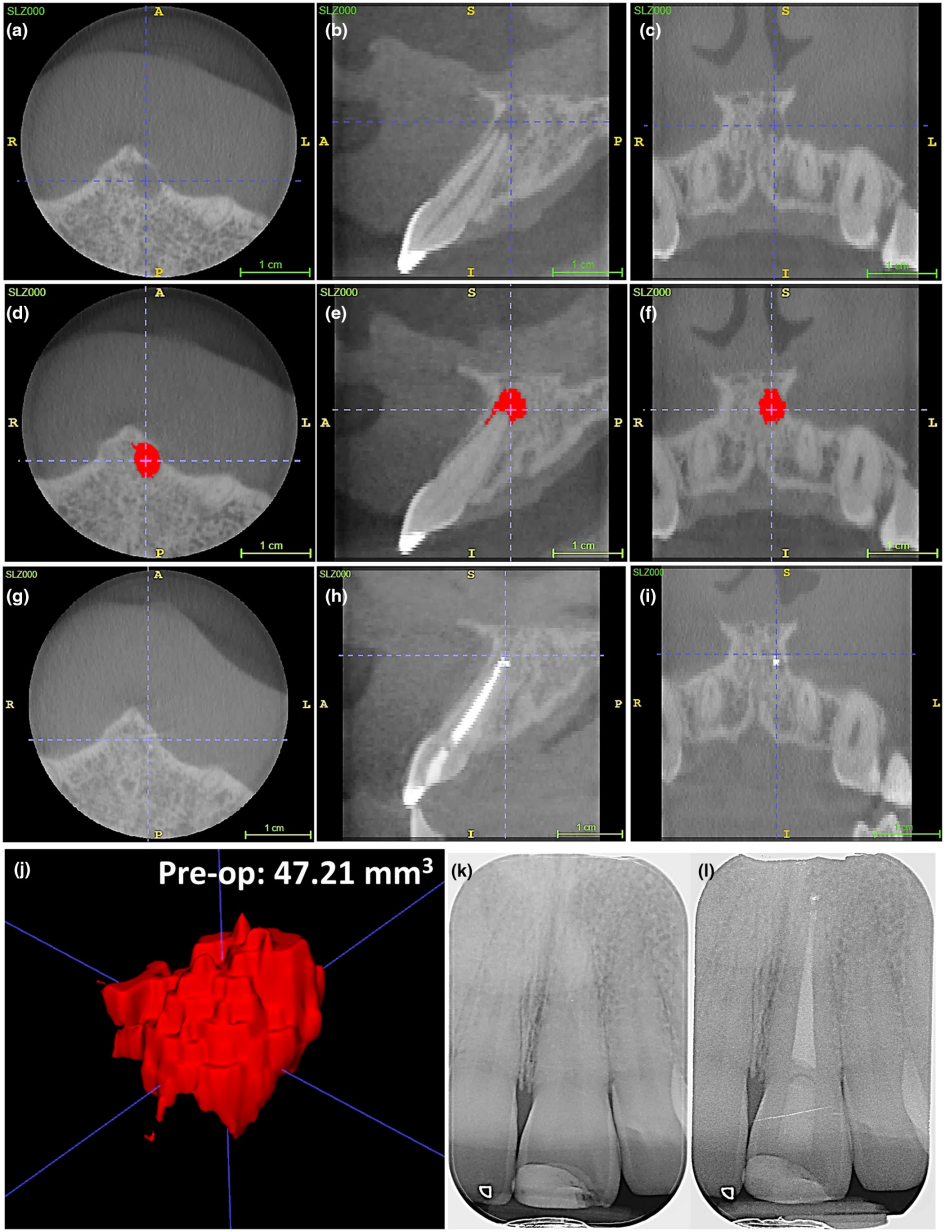
Longitudinal volumetric cone beam computed tomography analysis of periapical lesions following root canal treatment of the UL1. The example represents the axial [a,d,g], sagittal [b,e,h] and coronal [c,f,i] views alongside the preoperative volumetric analysis [j] of a lesion that has completely resolved following 17% EDTA irrigation at the 12 months review interval [g,h,i]. The corresponding pre‐ and postoperative periapical radiographs are depicted in [k] and [l], respectively. Scale bars represent 1 cm.

**FIGURE 5:**
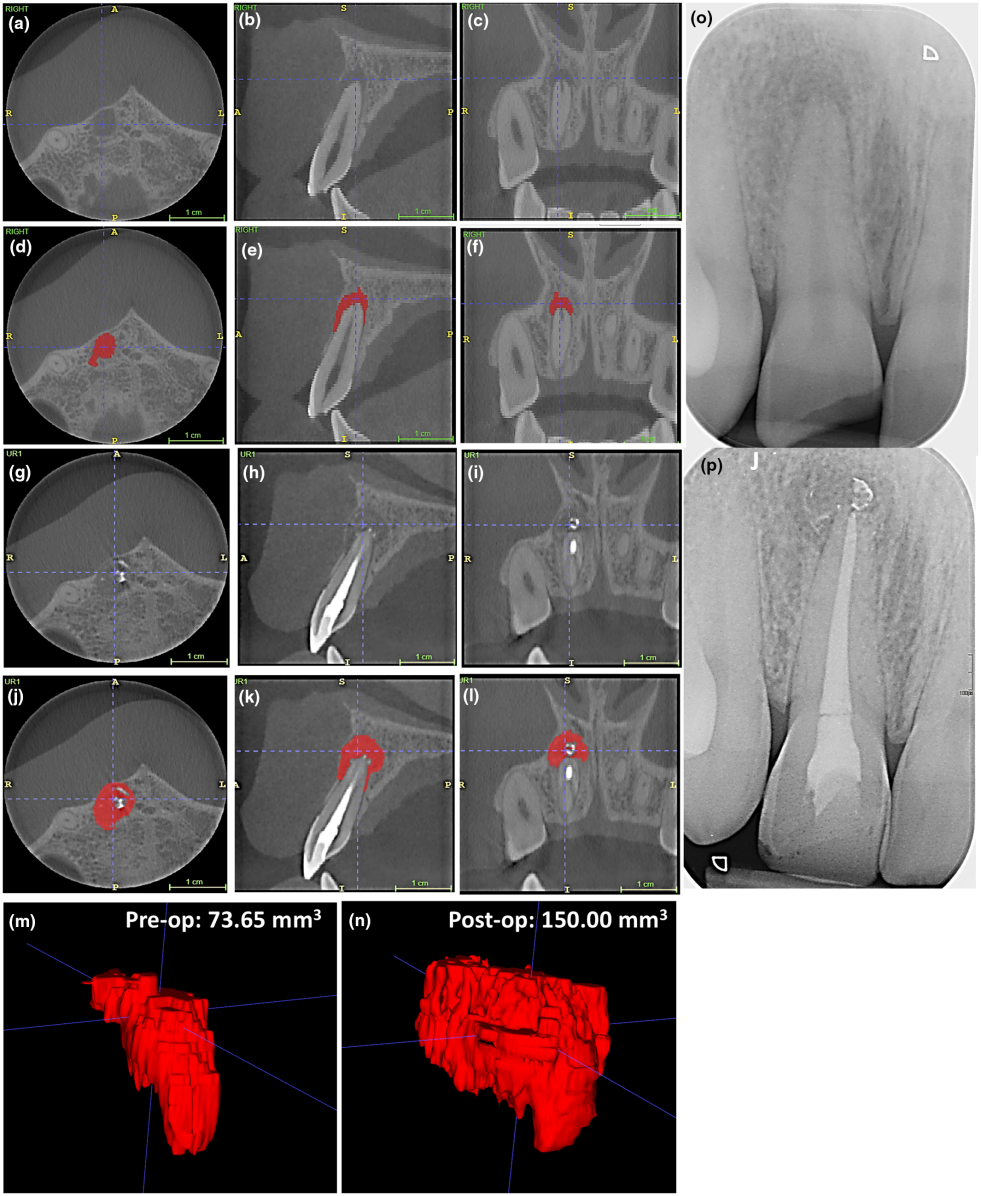
Longitudinal volumetric cone beam computed tomography analysis of periapical lesions following root canal treatment of the UR1. The example represents the axial [a,d,g,j], sagittal [b,e,h,k] and coronal [c,f,i,l] views alongside the pre‐ [m] and postoperative [n] volumetric analysis of a lesion that has increased in size following 5% NaOCl irrigation at the 12 months review interval [g–l]. The corresponding pre‐ and postoperative periapical radiographs are depicted in [o] and [p], respectively. Scale bars represent 1 cm.

**FIGURE 6:**
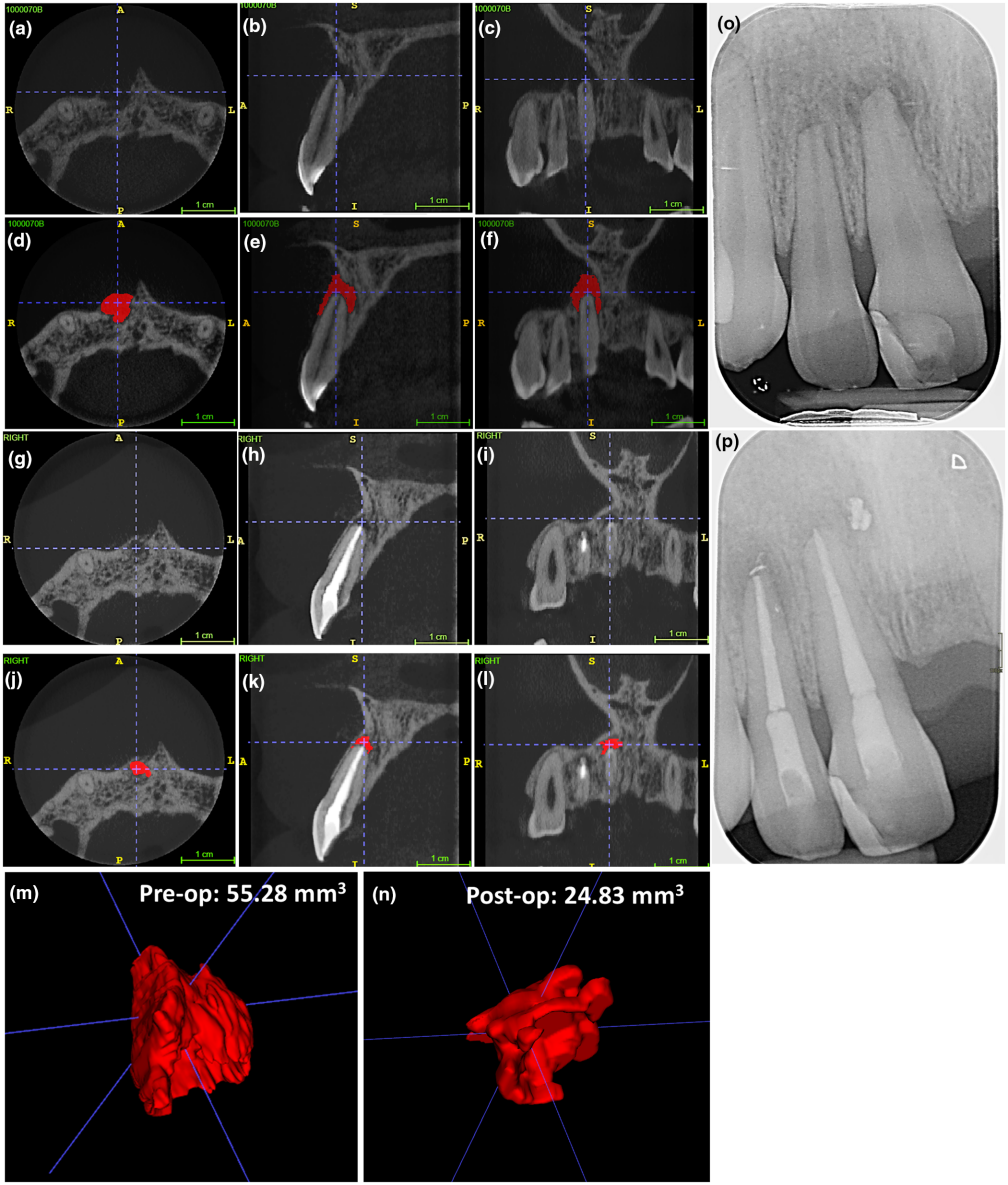
Longitudinal volumetric cone beam computed tomography analysis of periapical lesions following root canal treatment of the UR1. The example represents the axial [a,d,g,j], sagittal [b,e,h,k] and coronal [c,f,i,l] views alongside the pre‐ [m] and postoperative [n] volumetric analysis of a lesion that has reduced in size following 17% EDTA irrigation at the 12 months review interval [g–l]. The corresponding pre‐ and post‐operative periapical radiographs are depicted in [o] and [p], respectively. Scale bars represent 1 cm.

The intra‐ and inter‐rater agreements for determining periapical lesion volume exceeded 0.90, with results summarized in Table 4 and Figure 3 with representative examples of healed, healing and progressing lesions depicted in Figures 4, 5, 6, respectively. For the NaOCl group, the median pre‐ and postoperative periapical lesion volume was 78.9 mm^3^ [49.81–183.30] and 5.8 [2.75–32.70], respectively, resulting in a statistically significant percentage change of 92.50% [67.33–99.13] (*p* < .05). Similarly, the median pre‐ and postoperative lesion volume for the EDTA group was 66.8 [47.21–116.35] and 2.6 [0–13.17], respectively, giving a statistically significant percentage change of 95.84% [78.81–100] (*p* < .05). No significant differences were observed in these outcomes between groups (*p* > .05).

**TABLE 4:** Volumetric and percentage changes in periapical lesion size based on cone beam computed tomography scans of teeth diagnosed with asymptomatic apical periodontitis undergoing root canal treatment with conventional irrigant regimes and those that promote the release of dentine extracellular matrix proteins.

*n*	5% NaOCl			17% EDTA			*p* value^a^
	Preoperative lesion volume (mm^3^)	Postoperative lesion volume (mm^3^)	Percentage change in lesion volume (%)	Preoperative lesion volume (mm^3^)	Postoperative lesion volume (mm^3^)	Percentage change in lesion volume (%)	
1	22.85	0	100.00	6.90	2.63	95.84	
2	14.10	0	100.00	187.10	0	100.00	
3	7.34	0	100.00	45.22	75.82	78.81	
4	186.65	4.77	97.45	184.70	0	100.00	
5	44.415	15.05	66.12	188.10	0	100.00	
6	183.80	63.97	65.20	689.70	8.03	95.23	
7	187.10	0	100.00	74.20	0	100.00	
8	690.70	22.67	96.72	183.20	0	100.00	
9	75.55	74.64	1.21	26.56	0	100.00	
10	182.80	79.89	56.30	55.28	24.83	78.66	
11	25.86	4.51	82.54	333.60	10.82	90.88	
12	55.21	9.49	82.82	79.60	7.03	−138.63	
13	333.70	5.75	98.28	73.20	18.55	74.42	
14	78.93	24.83	68.54	81.50	0	100.00	
15	73.65	150.00	−103.67	160.70	13.17	80.27	
16	82.42	0	100.00	120.20	0	100.00	
17	161.55	40.57	74.89	63.30	24.32	50.93	
18	120.90	4.39	96.37				
19	64.20	4.81	92.50				
**Total**	78.9 [49.81–183.30]	5.8 [2.75–32.70]	92.50 [67.33–99.13]	66.8 [47.21–116.35]^b^	2.6 [0–13.17]^b^	95.84 [78.81–100]	.379

^a^ NaOCl vs. EDTA percentage change in lesion volume (*Mann–Whitney U* Test).

^b^ *p* < .05: preoperative vs. postoperative intra‐group comparison (Wilcoxon Matched Paired test Test); Total volumes and percentage changes are represented as medians [interquartile range].

## DISCUSSION

This is the first triple‐blinded parallel‐group randomized controlled phase I/II trial assessing the clinical effectiveness of endodontic irrigant regimes that enhance dECMs release. When compared with conventional antimicrobial approaches that utilize NaOCl, no significant difference in treatment outcomes could be observed at the 12‐month recall in single‐rooted teeth diagnosed with a necrotic pulp and asymptomatic apical periodontitis. This was based on loose (NaOCl:89.5%; EDTA: 94.1%) and strict (NaOCl: 26.3%; EDTA: 47.1%) criteria. Moreover, no statistical differences between groups were identified for patient‐reported pain scores; changes in periapical lesion volume and with the exception of IL‐6 and IL‐18, which were more abundant in the intervention group at the point of obturation. Irrigant regimes promoting dECM release thus demonstrated equivalence to conventional protocols with no adverse effects, and for this reason, we fail to accept the null hypothesis at the chosen level of significance.

The present study was designed to overcome many of the biases typically associated with interventional studies. More specifically, the robust randomization and concealment process facilitated statistical matching of participants across every demographic and clinical characteristic investigated. Patients, operators and assessors were all blinded, a feature that has inherently been difficult to implement in endodontic studies (Yi et al., 2020). Each of the prespecified outcomes in the protocol published *a priori* was reported, and recall rates of 90% and greater were achieved for the primary and secondary outcomes, respectively, which compares favourably with clinical studies with similar sample sizes and review intervals (Arslan et al., 2019; Martins et al., 2014; Xuan et al., 2018). Notably, the most common reason provided by those failing to attend, one of whom had been randomized into the test arm twice, was COVID‐19 travel restrictions, a factor that also led to a protracted recruitment and study period. Nevertheless, all patients barring the one deceased were contactable at the time of review and confirmed the teeth treated were still present, asymptomatic and functional, reducing the likelihood these would have contributed to failing outcomes. This rationalized the per‐protocol analysis that is commonly utilized in most other endodontic clinical trials and studies (Martins et al., 2014, Xuan et al., 2018, Arslan et al., 2019, Saini et al. 2022).

Results of the reported clinical trial, however, still need to be interpreted with caution due to several methodological limitations. Most notably, the limited sample size may result in an over‐ or under‐estimation of the effects. A smaller cohort was selected as part of phase I/II trial here as the safety and efficacy of the proposed irrigant regime have not been established in combination with the conventional understanding that EDTA, predominantly known for its chelating action on smear layers and antifungal properties, lacks robust antibacterial activity (Arias‐Moliz et al., 2008; Arias‐Moliz et al., 2009; de Almeida et al., 2016; Mohammadi et al., 2013; Ordinola‐Zapata et al., 2012; Siqueira Jr et al., 1998). Whilst limited evidence pertains to an antibiofilm effect, it is currently unknown if this is sufficient to clinically promote the endodontic micro‐environment necessary to facilitate periradicular tissue healing (Giardino et al., 2020; Siqueira Jr & Rôças, 2008; Virdee et al., 2023a). Consequently, this underpinned the rationale for a two‐visit approach; as the inter‐appointment calcium hydroxide period, which aimed to compensate for an absence of NaOCl via its organic solvent and antimicrobial properties, gave researchers the opportunity to revert back to conventional irrigant regimes if severe side effects were encountered. Furthermore, this medicament provided the added benefit of not deleteriously affecting the integrity of dECMs (Graham et al., 2006 and Tomson et al., 2017). Nevertheless, future studies with increased sample sizes are now required to strengthen the reported findings.

Single‐rooted teeth diagnosed with a necrotic pulp and asymptomatic apical periodontitis were selected as they improved standardization of endodontic anatomy and in turn the contact surface area available for dECM solubilization, reducing operator error risk and simplifying the PTF sampling process (Vertucci, 1984; Virdee et al., 2023b). As there is also no prior clinical safety data on the use of EDTA as a sole irrigant, single‐rooted teeth were determined to be more amenable to remedial therapy in the event of unexpected adverse events during or after treatment. Furthermore, root canals with previously initiated treatment will have been subjected to unknown irrigant regimes that could have inadvertently reduced dECM bioavailability (Virdee et al. [submitted for publication]), whereas those that were root treated or symptomatic are associated with differing endodontic microbiomes, inflammatory pathophysiology and treatment outcomes; all of which may have influenced results (Galler et al., 2015; Martinho et al., 2016; Ng et al., 2011; Siqueira Jr & Rôças, 2022). Whilst this may have improved internal validity, it will have limited generalizability, particularly in a secondary care setting where these teeth are seldom encountered. Further investigations are required to determine if similar outcomes are observed in multi‐rooted teeth, where irrigation protocols may play a greater role in treatment success, or those that present with more acute clinical signs and symptoms of periradicular disease (Laukkanen et al., 2021).

In the current study, favourable outcomes were observed in 89.5% of participants who underwent conventional irrigation, which is consistent with many other analogous reports (Liang et al., 2014; Ng et al., 2011; Yildiz et al., 2024). Surprisingly, a slightly higher but statistically insignificant result of 94.1% was observed in the EDTA group, for which there are currently no comparable clinical investigations. These outcomes were derived from robust measures that included a thorough clinical examination, highly sensitive volumetric imaging techniques and guideline standard recall intervals (ESE 2006, Lofthag‐Hansen et al., 2007, Duncan et al., 2021). It is acknowledged, however, that a 12‐month review period may be too short to adequately assess for complete periradicular healing, a process that, whilst occurring over two years in most cases, can reportedly take up to four (Ørstavik 1996, Ng et al., 2011). This would account for the considerably lower success rates found in both arms, which were still, nevertheless, statistically comparable.

The high rates of favourable healing in the intervention group could be explained by the mechanisms proposed in the original hypothesis. For instance, a recent in vivo observational study confirmed endogenous TGF‐β1 within the root canals of mature permanent teeth following ultrasonically activated EDTA irrigation, following the use of a similar regime that was applied in the present study (Widbiller et al., 2022). Moreover, these were detected at quantities previously shown to elicit tissue healing responses; however, NaOCl irrigation significantly hampered these processes (Galler et al., 2015 and 2016). Although a narrow minor apical foramen may limit the periapical interface, in vitro experiments conducted by the authors provide proof of concept for adequate periradicular bioavailability of dECMs when this structure was only minimally enlarged as per the above in vivo protocol (i.e. 0.2 mm; Virdee et al. [submitted for publication]). More specifically, methylcellulose strips were externally adapted to the apical thirds of intact extracted teeth during EDTA irrigation of standardized root canals and found to have absorbed bioactive quantities of TGF‐β1 when analysed using enzyme‐linked immunosorbant assays (Virdee et al. [submitted for publication]). This contrasts with previous recommendations of pre‐enlarging the apical foramen to 0.5–1.0 mm and can be attributed to this size being based on the need for intraradicular influx of sufficient blood and cellular components, which require a larger interface than for the dECMs molecules (Fang et al., 2018; Kim et al., 2018). Finally, the regenerative effects dECMs have on the various endodontic MSC niches are well documented (Virdee et al., 2022), with the authors being able to also demonstrate upregulation of proliferation, chemotaxis, osteogenic differentiation and calcific mineralization in human PL‐MSC cultures (Virdee et al. [submitted for publication]). This collective body of evidence corroborates the proposed hypothesis.

Another implication of this work is that chemomechanical preparation with 17% EDTA provided the endodontic disinfection necessary to initiate periradicular regeneration on par with 5.25% NaOCl irrigation. This outcome could be explained by its chelating properties that destabilizes the outer cell membranes of gram‐negative bacteria (Finnegan & Percival, 2015). Whilst this effect alone may not always induce bacterial death it could be sufficient when combined with mechanical instrumentation (Virdee et al., 2023a). The chelating action has also been shown to promote cellular detachment and weaken the macrostructures of established biofilms, which can then be more easily flushed from root canals via the mechanical shearing forces created by conventional irrigant flow dynamics and agitation techniques (Bryce et al., 2009; de Almeida et al., 2016). Future clinical studies could go some way to investigate this by longitudinally quantifying the endodontic bacterial load and endotoxin levels in both groups. It must be acknowledged however that the extended duration of EDTA exposure to dentine with the proposed irrigant regime far exceeds current recommendations and may increase dentinal corrosion and reduce the dentine' microhardness (Niu et al., 2002; Saleh & Ettman, 1999). Whether this clinically translates into increased risks of vertical root fractures or reduced rates of periapical healing however has yet to be demonstrated.

Many of the single‐rooted teeth that were recruited into the trial developed periradicular lesions following traumatic dental injuries. When sampled, the endodontic microflora in these circumstances has been demonstrated to be significantly different to that in root canals infected for nontraumatic reasons with respect to composition and diversity (Manoharan et al., 2020). More specifically, there is a higher predominance of anaerobic species belonging to the genera *Bacteroides, Corynebacterium, Peptostreptococcus* and *Fusobacterium* (Fouad, 2019; Manoharan et al., 2020). These variations may make the microflora more susceptible to chemomechanical disinfection protocols irrespective of the irrigant used and could alternatively explain the results of the present study. Expanding the inclusion criteria to include multi‐rooted teeth, which are often referred to secondary care settings, would address this selection bias. Other explanations that require further investigation could be attributed to the anatomical simplicity of single‐rooted teeth that may not thoroughly test the efficacy of irrigants in all tooth types; asymptomatic lesions, which have a less virulent microbiota; the two‐week intracanal medicament period with calcium hydroxide or underpowered sample size (Laukkanen et al., 2021; Siqueira Jr & Rôças, 2022).

The Target‐48 panel consistently detected 15 PTF‐derived cytokines (CCL‐2, −3, −4; CSF‐1; CXCL‐8; HGF; IL‐1β, −6, −18; MMP‐1, −12; OLR‐1; OSM; TNFSF‐10; VEGF‐A) all of which have previously been associated with periradicular inflammation (Márton & Kiss, 2014). Indeed, these were detected in several immunohistochemical investigations and a cross‐sectional PTF study that, through similar methodologies, also identified CXCL‐9, IL‐17A and TNFSF‐12 (Kabashima et al., 2002; Nonaka et al., 2008; Tsai et al., 2008; Virdee et al.,  2023b). In the current analyses, these three molecules were marginally below the 75% inclusion threshold, which was unexpected for the former, as this was deemed the most diagnostically distinguishable biomarker for asymptomatic apical periodontitis (Appendix A; Virdee et al., 2023b). As this discrepancy is likely due to the relatively smaller sample size, there was reluctance to proceed with further receiver operating characteristic analyses with respect to treatment outcomes. These data, nevertheless, provide useful insight into periradicular pathophysiology, as future investigations can focus on the roles these cytokines play in the disease process. Particular attention should be given to IL‐6 and IL‐18, which, although reduced in concentration throughout treatment, were still significantly more abundant in the test group prior to obturation. Interestingly, whilst the former cytokine is considered pro‐inflammatory, it has been associated with inhibiting IL‐1β and TNF‐α induced bone resorption, which results in larger periapical lesions, and has potent regenerative activities in the liver and kidneys (Balto et al., 2001; Galun & Rose‐John, 2013; Kwan Tat et al., 2004).

Given the lack of prior safety data on the proposed irrigant regime, patient‐reported pain scores were considered an important outcome measure by the public involvement group. The NRS was thus used as it is significantly simpler and more sensitive than its visual analogue and verbal rating scale counterparts (Hjermstad et al., 2011). For post‐obturation, this tool revealed similar results for both groups, with pain profiles being highly consistent with those typically described in multiple analogous reports. More specifically, it was of mild severity, peaking within 24 h and lasting only one to two days, where it would then taper down to negligible levels by one week (Sathorn et al., 2008, Pak & White, 2011, Ballal et al., 2019). For post‐instrumentation, whilst the incidences of discomfort were comparable between groups, it was conventional irrigant regimes that were associated with notably more severe pain profiles and greater use of analgesics; albeit this outcome was not statistically significant. Additionally, the only two acute exacerbations, which nevertheless went on to experience favourable healing outcomes, occurred in this group. Notably, similar characteristics have been observed in the relatively few investigations that have explored inter‐appointment pain in teeth with a necrotic pulp (Mostafa et al., 2020; Ng et al., 2011). This finding can be attributed to NaOCl's cytotoxic nature, which is a function of its concentration, with inflammatory effects on periapical tissues lasting up to 30 days following administration (Gomes‐Filho et al., 2008). Furthermore, the efflux of highly concentrated NaOCl into the periradicular region is likely to have been exacerbated by the intentional pre‐enlargement of the apical foramen (Tinaz et al., 2005). Therefore, based on the present findings, no adverse effects or pain profiles were observed for the aforementioned EDTA‐derived irrigant regime.

## CLINICAL IMPLICATIONS

This triple‐blinded randomized controlled clinical study provides preliminary data suggesting the healing rates of periapical lesions with irrigation regimes aimed at releasing bioactive dECMs are comparable to conventional disinfection protocols. This is supported by in vitro mechanistic data that demonstrates the efficacy of this irrigant regime to release dECMs and their subsequent bioavailability to periradicular tissues in mature permanent teeth and regenerative effects on local MSCs (Virdee et al. [submitted for publication]). As favourable outcomes were observed on par with 5.25% NaOCl treatment, it can be inferred that the bacterial load was reduced beyond the critical threshold necessary to facilitate periradicular healing (Siqueira Jr & Rôças, 2008). This brings into question the accepted approach of solely disinfecting the root canal system to treat apical periodontitis and not including other therapeutic strategies and mechanisms to enhance the healing of diseased periradicular tissues. The reliance on NaOCl treatment may have thus masked a previously undetected biological mechanism to treat apical periodontitis, given its deleterious effects on dECMs (Galler et al., 2015, Virdee et al. [submitted for publication]). Strategies that go beyond simply disinfecting root canals and which exploit innate healing mechanisms as proposed may thus improve treatment outcomes and reduce reliance on NaOCl, which when extruded can lead to severe swellings or neuroanatomical damage (Farook et al., 2014). Such approaches may lead to a paradigm shift for treating apical periodontitis. The present findings do however require further exploration via appropriately powered phase III trials with longer review intervals and wider inclusion criteria. It is also well established that prior to any tissue engineering protocol, a pro‐healing environment is a mandatory pre‐requisite (Kim et al., 2018). This could be considered as one where the endodontic bacterial load is below that necessary to initiate periradicular healing events, a threshold that is currently yet to be defined (Siqueira Jr & Rôças, 2008).

## CONCLUSION

In this triple‐blinded randomized controlled phase I/II trial of single‐rooted necrotic mature permanent teeth, therapeutic irrigant regimes promoting dECMs solubilization exclusively with 17% EDTA resulted in favourable one‐year treatment outcomes that were equivalent to conventional 5.25% NaOCl‐based irrigant regimes. Moreover, no severe adverse effects or atypical pain profiles were observed in the former, demonstrating the clinical safety of 17% EDTA. Longitudinal analysis of PTF‐derived biomarkers revealed significant reductions in the various inflammatory cytokines that were common for both groups, although, interestingly, IL‐6 and IL‐18 were significantly more abundant in the intervention group. Further investigations with a larger sample size and wider inclusion criteria are nevertheless warranted to strengthen the findings of this clinical approach and delve deeper into the profile of inflammatory biomarkers found in PTF, which will lead to a better understanding of prognostic indicators for treatment of apical periodontitis.

## AUTHOR CONTRIBUTIONS

**Satnam Singh Virdee**: Conceptualization (equal); data curation (lead); formal analysis (supporting); investigation (lead); methodology (equal); project administration (equal); resources (supporting); validation (supporting); visualization (lead); writing–original draft preparation (lead); writing–review and editing (equal). **Nasir Zeeshan Bashir**: Conceptualization (equal); data curation (supporting); formal analysis (Lead); investigation (supporting); methodology (supporting); validation (lead); visualization (supporting); writing–original draft preparation (supporting); writing–review and editing (equal). **Melissa M. Grant**: Conceptualization (equal); data curation (supporting); formal analysis (supporting); methodology (equal); project administration (equal); resources (lead); supervision (equal); writing–review and editing (equal). **Paul R. Cooper**: Conceptualization (equal); supervision (equal); writing–review and editing (equal). **Phillip L. Tomson**: Conceptualization (lead); methodology (equal); funding acquisition (lead); supervision (equal); writing–review and editing (equal); investigation (Chief).

## FUNDING INFORMATION

2017‐BES Annual Research Grant.

## CONFLICT OF INTEREST STATEMENT

The authors deny any conflicts of interest related to this study.

## Data Availability

The data that support the findings of this study are available from the corresponding author upon reasonable request.

## APPENDIX A Complete Target‐48 Panel with missing data frequency (%) and raw concentration values (pg/mL) for analytes that met the detection threshold

### A.1

**Table:**

Target 48 Analyte	Missing Data (%)	Raw Analyte Concentration (pg/mL)			
		Baseline (S1)		Pre‐obturation (S2)	
		5% NaOCl	17% EDTA	5% NaOCl	17% EDTA
Chemokine Ligand [CCL]‐2	3	8.5 [2.30–16.34]	10.3 [5.36–66.88]	1.02 [0.16–7.47]	2.1 [0.59–10.83]
Chemokine Ligand [CCL]‐3	5	7.6 [0.97–17.38]	7.4 [2.23–18.67]	0.5 [0.16–2.01]	0.5 [0.13–2.51]
Chemokine Ligand [CCL]‐4	8	19.7 [4.34–52.53]	27.0 [7.25–50.46]	1.1 [0.40–5.43]	1.78 [0.13–10.07]
Chemokine Ligand [CCL]‐7	70	–	–	–	–
Chemokine Ligand [CCL]‐8	50	–	–	–	–
Chemokine Ligand [CCL]‐11	93	–	–	–	–
Chemokine Ligand [CCL]‐13	73	–	–	–	–
Chemokine Ligand [CCL]‐19	95	–	–	–	–
Chemokine Ligand [CXCL]‐9	35	–	–	–	–
Chemokine Ligand [CXCL]‐10	70	–	–	–	–
Chemokine Ligand [CXCL]‐11	93	–	–	–	–
Chemokine Ligand [CXCL]‐12	100	–	–	–	–
Colony Stimulating Factor [CSF]‐1	10	1.6 [0.42–2.51]	1.5 [0.70–3.41]	0.1 [0–0.97]	0.3 [0.14–0.87]
Colony Stimulating Factor [CSF]‐2	93	–	–	–	–
Colony Stimulating Factor [CSF]‐3	55	–	–	–	–
Epidermal Growth Factor [EGF]	90	–	–	–	–
Fms‐related Tyrosine Kinase 3 Ligand [FLT3LG]	90	–	–	–	–
Hepatocyte Growth Factor [HGF]	3	41.7 [11.94–109.66]	62.7 [31.10–102.17]	2.7 [0.48–7.44]	8.2 [0.50–33.24]
Interferon Gamma [IFNG]	78	–	–	–	–
Interleukin [IL]‐1β	3	70.0 [23.93–185.92]	146.28 [47.27–249.04]	0.5 [0.11–1.59]	0.8 [0.40–4.27]
Interleukin [IL]‐2	100	–	–	–	–
Interleukin [IL]‐4	100	–	–	–	–
Interleukin [IL]‐6	5	2.8 [0.77–6.35]	1.7 [0.69–4.84]	0.1 [0.02–0.44]	0.7 [0.10–6.07]
Interleukin [IL]‐7	100	–	–	–	–
Interleukin [CXCL]‐8	3	271.6 [46.02–1254.32]	709.0 [53.60–1953.12]	16.5 [3.83–225.94]	23.9 [3.97–312.31]
Interleukin [IL]‐10	78	–	–	–	–
Interleukin [IL]‐13	93	–	–	–	–
Interleukin [IL]‐15	100	–	–	–	–
Interleukin [IL]‐17A	35	–	–	–	–
Interleukin [IL]‐17C	100	–	–	–	–
Interleukin [IL]‐17F	90	–	–	–	–
Interleukin [IL]‐18	0	41.0 [19.66–91.86]	54.9 [34.18–81.15]	0.2 [0.19–4.52]	13.78 [1.46–41.45]
Interleukin [IL]‐27	100	–	–	–	–
Interleukin [IL]‐33	90	–	–	–	–
Lymphotoxin [LT]‐α	93	–	–	–	–
Matrix Metallopeptidase [MMP]‐1	20	18.7 [1.53–67.80]	10.80 [2.03–60.40]	0.5 [0–14.76]	1.8 [0.02–21.06]
Matrix Metallopeptidase [MMP]‐12	20	265.9 [12.94–2222.72]	143.32 [22.44–547.28]	41.4 [4.38–125.25]	26.1 [1.54–94.9]
Oncostatin M [OSM]	8	9.7 [2.05–29.18]	15.3 [5.83–39.55]	0.19 [0.06–3.06]	0.23 [0.04–3.63]
Oxidised Low Density Lipoprotein Receptor [OLR]‐1	5	694.0 [155.40–1020.85]	804.6 [414.41–931.45]	11.2 [2.05–115.58]	11.16 [0.73–208.13]
Thymic Stromal Lymphopoietin [TSLP]	95	–	–	–	–
Transforming growth factor [TGF]‐α	63	–	–	–	–
Tumour Necrosis Factor [TNF]	80	–	–	–	–
Tumour Necrosis Factor Superfamily [TNFSF]‐10	8	15.1 [4.41–39.22]	27.2 [14.06–36.85]	0 [0–0.57]	0 [0–2.86]
Tumour Necrosis Factor Superfamily [TNFSF]‐12	33	–	–	–	–
Vascular Endothelial Growth Factor [VEGF]‐A	3	40.0 [15.36–79.90]	63.7 [32.51–90.28]	4.4 [1.07–25.02]	6.9 [2.27–20.84]
